# The Effects of Lowering Uric Acid Levels Using Allopurinol on Components of Metabolic Syndrome

**DOI:** 10.4021/cr168w

**Published:** 2012-03-20

**Authors:** Esther J. Heimbach, Rodney G. Bowden, Jackson O. Griggs, A. Alexander Beaujean, Eva I. Doyle, Robert D. Doyle

**Affiliations:** a Baylor University, Waco, Texas, USA; b Family Health Center, Waco, Texas, USA

**Keywords:** Cholesterol, LDL, HDL, Hyperuricemia, Gout

## Abstract

**Background:**

Researchers have reported an independent direct relationship between lipid levels and hyperuricemia with MetS. The purpose of this study was to determine the relationship between serum uric acid levels and lipids among patients on allopurinol.

**Methods:**

A retrospective secondary data analysis was conducted on 66 adult patients from a family health clinic in Central Texas. Medical records used were recorded during a nine year period (2002 - 2010) ascertaining the relationship between uric acid and lipids.

**Results:**

Spearman correlations revealed a weak correlation between uric acid and total cholesterol, a weak correlation between uric acid and triglycerides and LDL-C. A weak inverse correlation was discovered between uric acid and HDL-C. A moderate correlation was discovered when all lipid variables combined were compared to uric acid.

**Conclusions:**

We discovered LDL-C and triglycerides to be significant predictors of uric acid with weak correlations. Additionally, weak correlations existed between uric acid and total cholesterol and HDL-C with an inverse relationship discovered with HDL-C. These findings support the literature suggesting that uric acid is more likely to be associated with total cholesterol and triglycerides. In addition, new discoveries serve as an indication that LDL-C may also be associated with uric acids levels. The mechanism by which uric acid may regulate lipids is elusive but suggestions have included suppression of lipid peroxidase and decreases in critical lipase activity.

## Introduction

Metabolic Syndrome (MetS) is a significant public health issue in the United States, affecting 27-34% of adults [1, 2]. MetS increases the adult risk of chronic kidney disease [3], type 2 diabetes [4], and mortality from cardiovascular disease (CVD) and coronary heart disease (CHD) [5] and is characterized by a clustering of conditions that vary depending upon the organizational definition. The World Health Organization, the National Cholesterol Education Program- Third Adult Treatment Panel [6] and the International Diabetes Federation agree that the core components of MetS include obesity, insulin resistance, dyslipidemia, and hypertension [7]. However, a strong association between hyperuricemia and MetS has been reported [8] leading some researchers to conclude that uric acid levels should be considered in the diagnosis of MetS [9, 10].

Not only is hyperuricemia closely related to the diagnosis of MetS, a relationship exists between hyperuricemia and the particular features of MetS. An independent direct relationship exists between lipid levels and hyperuricemia in MetS patients [11-14]. Accordingly, high density lipoprotein cholesterol (HDL-C) negatively correlates with hyperuricemia, whereas triglycerides positively correlate [11-13]. Additionally, waist circumference, body mass index (BMI) and blood pressure show similar positive correlations with hyperuricemia [11, 12].

The expectation that lowering uric acid levels would decrease the occurrence of MetS, though, has not been supported by findings that indicate a more complex relationship between uric acid lowering agents and the components of metabolic syndrome. Hyperuricemia was correlated with other components of MetS in one study such as blood pressure, obesity, and immunoreactive insulin, but did not correlate with components such as leptin or blood glucose levels [12]. Yet, specifically hyperuricemia has most significantly correlated with triglyceride levels and BMI in other studies [12-13]. Conversely, another study [15] suggested that high doses of fructose induced features of MetS, but allopurinol did not reduce fasting plasma triglyceride levels. Finally, a study among end-stage renal disease patients [16] determined that lowering serum uric acid levels with allopurinol elevated triglyceride levels, although it decreased low density lipoprotein (LDL-C). The association between uric acid and lipid levels is further complicated by the findings from Suliman et al [17] that indicated a “J-shaped” association with all-cause mortality and in studies using animal models [18, 19]. Therefore, the purpose of this study was to determine the relationship between serum uric acid levels and lipids among patients on allopurinol.

## Methods

A retrospective secondary data analysis was conducted among 66 adult patients from a family health clinic in central Texas with Metabolic Syndrome and gout as diagnosed by their treating physician. Medical records used were recorded during a nine year period (2002 - 2010). Data was extracted for patients who meet the following criteria: (a) age ≥18 years; and (b) received ≥3 allopurinol prescriptions between 2002 and 2010. Data used in the analysis included lipid variables (LDL-C, HDL-C, triglycerides, total cholesterol), and uric acid. Demographic information (ethnicity, gender, age) and the number of prescriptions of allopurinol were collected. During the study period venous blood samples (20 mL) were collected by a phlebotomist from each participant after a 12-hour fast using a standardized venipuncture technique in the antecubital vein for a standard lipid and uric acid panel. Venous samples, intended for serum analysis, were centrifuged and immediately placed in a cold storage unit and sent for assay. Lipid profiles were assessed using gel electrophoresis with whole blood samples sent to Quest Diagnostics (Dallas, Texas). Results of the serum analysis and lipid profiles were collected and entered into the patient records within the Epic Systems database management system. Approval was obtained by the appropriate university and clinic IRB groups.

### Statistical analysis

Spearman correlations were calculated to ascertain relationships between uric acid and all lipid variables. Additionally, scatterplots were created for each lipid variable in comparison to uric acid. Descriptive statistics were calculated for demographic variables. Statistical analyses were performed using SPSS software version 18.0 (IBM, Armonk, New York). All data is presented as mean ± standard deviation (SD) with P < 0.05 considered significant.

## Results

Demographic information for participants is presented in Table 1.

**Table 1:** Demographic Information for the Study Sample

Gender (n, %)		
Females	31	47.0
Males	35	53.0
Age (y, SD)	66	13.95
Ethnicity (n, %)		
African American	38	57.6
Caucasian	25	37.9
Hispanic	3	4.5
Uric Acid (mean, SD)	7.54	2.31
HDL-C (mean, SD)	45.23	11.01
LDL-C (mean, SD)	102.28	37.23
Total Cholesterol (mean, SD)	186.94	43.12
Triglycerides (mean, SD)	195.12	127.80

All measurement given in mg/dL.

**Figure 1:**
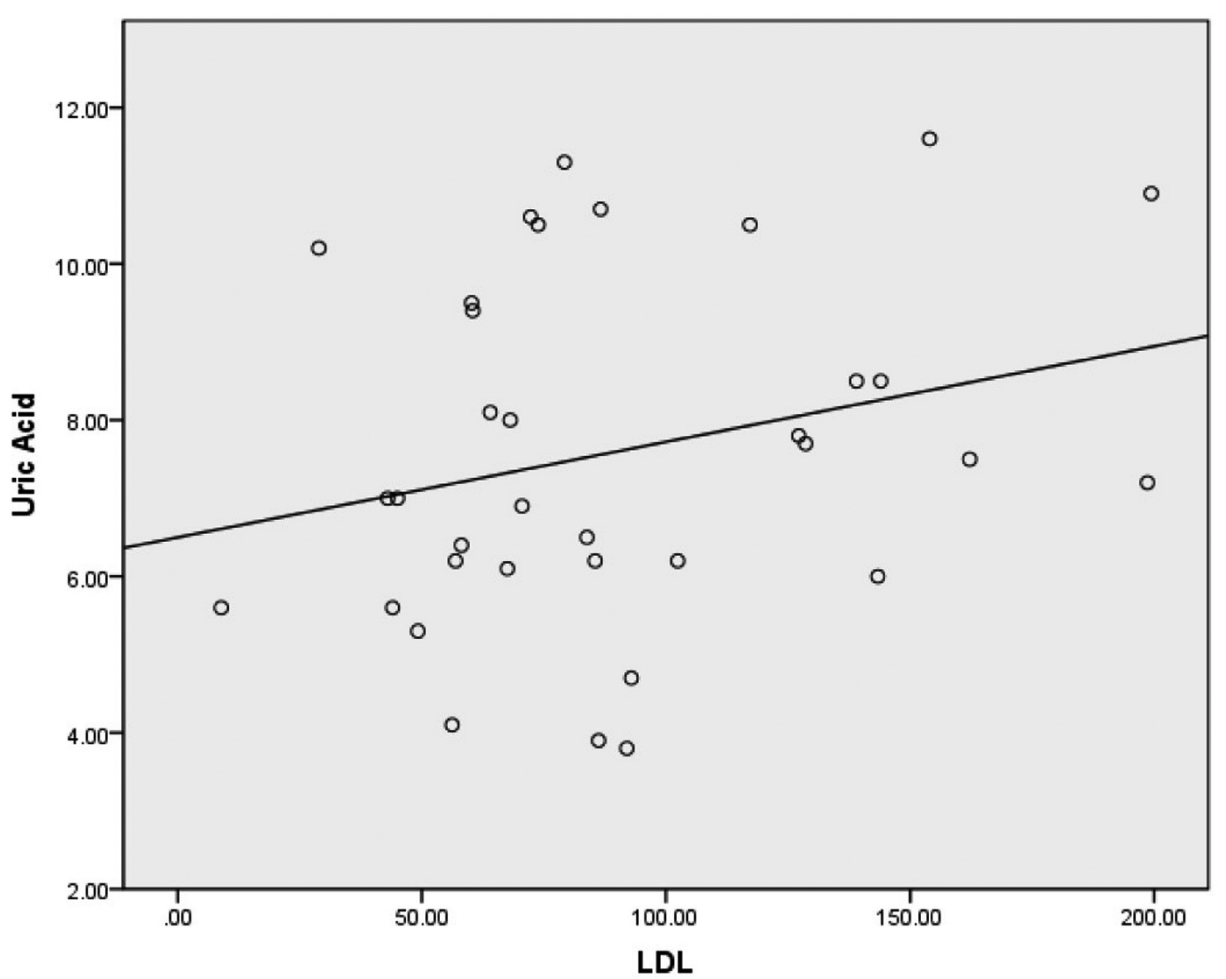
Scatterplot with a least squares regression line for LDL-C and uric acid.

**Figure 2:**
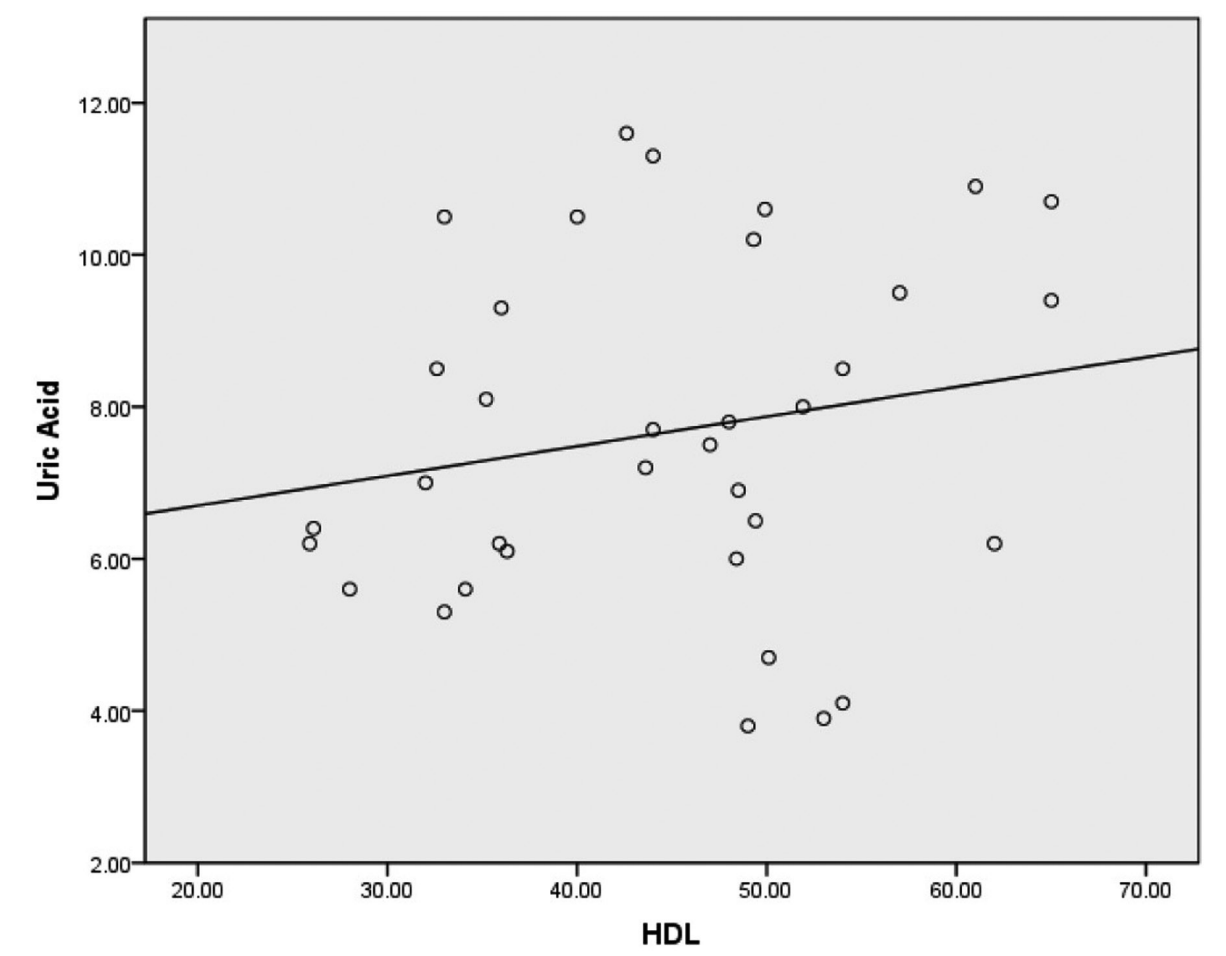
Scatterplot with a least squares regression line for HDL-C and uric acid.

**Figure 3:**
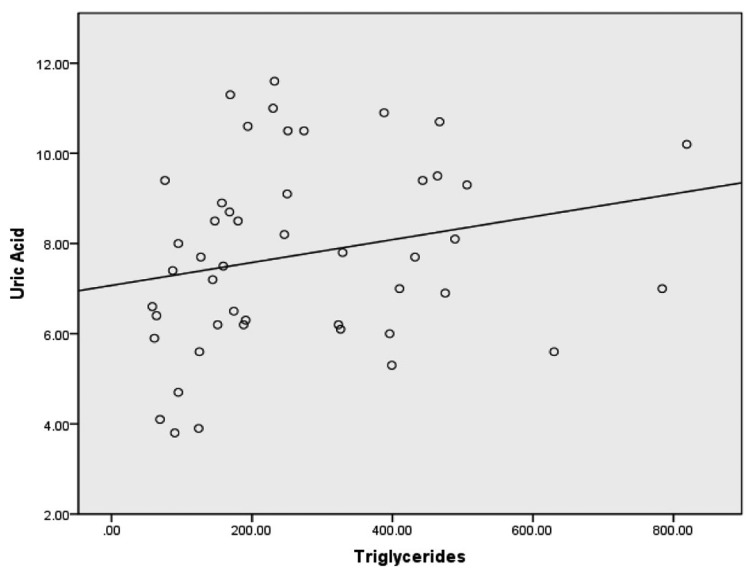
Scatterplot with a least squares regression line for triglycerides and uric acid.

**Figure 4:**
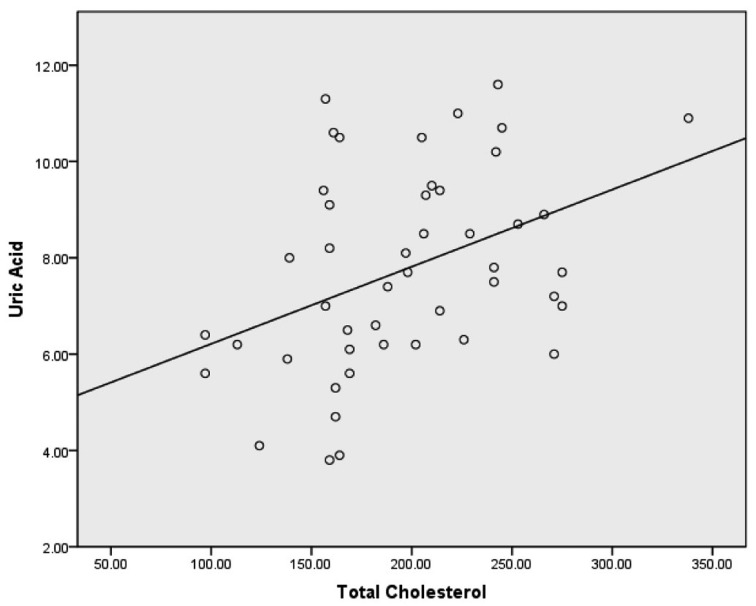
Scatterplot with a least squares regression line for total cholesterol and uric acid.

Spearman correlations revealed weak correlations between uric acid and total cholesterol, uric acid and triglycerides and uric acid and LDL-C. A weak inverse correlation was discovered between uric acid and HDL-C. A moderate correlation was also discovered when all lipid variables combined were compared to uric acid. Spearman correlations with corresponding bootstrapped 95% confidence intervals are presented in Table 2. Scatterplots for uric acid and each lipid variable are provided in Figure 1-4.

**Table 2:** Spearman Correlations With Corresponding Bootstrapped 95% Confidence Intervals Between Lipids and Uric Acid

Variable	Spearman Correlation	95% Confidence Interval
Total Cholesterol	0.293	0.004 - 0.580
Triglycerides	0.264	-0.177 - 0.487
HDL	-0.211	-0.585 - 0.242
LDL	0.192	-0.149 - 0.596
All lipid variables combined	0.418	-0.102 - 0.807

## Discussion

MetS is a significant public health issue that is associated with many comorbid conditions [3-5] and has an increasing prevalence rate among adults in the U.S. [1, 2]. Hyperuricemia has been linked with MetS [9, 10] and with criteria of MetS including glucose, insulin, blood pressure, obesity, waist circumference, waist-hip ratio, BMI, and lipid levels [11-13, 15, 20]. However, manipulation of uric acid levels among rats [18, 19] and humans [15, 16] suggest equivocal findings regarding the relationship of uric acid modification and lipid levels. In order to determine the best methods for managing MetS, research is needed that clarifies how uric acid modification affects lipid levels and ultimately cardiovascular outcomes, in patients with MetS [21]. Most studies have focused on risk factors for metabolic syndrome, but few have attempted to elucidate the relationship between uric acid and lipids [17]. Therefore, the purpose of this study was to determine the relationship between serum uric acid levels and lipid levels among patients on allopurinol.

Our study discovered that modified uric acid levels predict LDL-C and triglyceride levels with weak correlations. Additionally, weak correlations existed between uric acid and total cholesterol and HDL-C with an inverse relationship discovered with HDL-C. This finding support the literature suggesting that uric is more likely to be associated with triglycerides, but adds novel findings to the literature suggesting LDL-C may also be associated with uric acid levels.

The mechanism by which uric acid may regulate lipids is elusive but suggestions have included suppression of lipid peroxidase [22] and decreases in critical lipase activity [19]. Supportive of these theories is the findings of our study suggesting uric acid levels are associated with LDL-C and triglycerides. The association between triglycerides and uric acid in our study may be due to the function of lipoprotein lipase (LPL), a primary enzyme associated with both the lipolysis of triglycerides and the oxidation of LDL-C [23]. A decrease in LPL and its corresponding enzymatic activity has been reported to be associated with an increase uric acid [19, 21, 24]. The decrease in LPL has a downstream effect of oxidative stress, and reduced nitrous oxide release which may cause an increase in both LDL-C and triglycerides and is supported by our findings [21]. Furthermore, when hyperuricemia is combined with oxidized LDL-C there could be an increase in triglyceride levels [16]. Shelmadine et al [16] and Perez-Pozo et al [15] support our findings suggesting a significant relationship between LDL-C and uric acid through prospective studies involving the administration of allopurinol among participants. Additionally, studies[11-13] with human models and animal models [18-19] report a significant direct relationship between uric acid and triglycerides.

An unusual finding in our study was that uric acid was not a good predictor of total cholesterol. Previous study authors [25-26] have suggested that hyperuricemia may impair endothelium dependent vasodilatation via lipid oxidation which normally is thought to be associated with increases in total cholesterol. Animal models used in Balasubramanian [18] reported a significant direct relationship between uric acid levels and total cholesterol levels after the urinary bladders of rats were perfused with either uric acid or 1-methl uric acid. However, two other studies [12, 20] using animal models did not report a significant correlation between total cholesterol and uric acid levels. Our study supports the latter findings in that total cholesterol was not a significant predictor of uric acid levels.

Finally, uric acid was not identified as a strong predictor of HDL-C in our study. Shelmadine et al [16] reported a non-significant trend between uric acid and increasing levels of HDL-C in kidney failure patients. However, another study [12] reported a significant relationship between hyperuricemia and HDL-C in non-kidney disease patients. Most patients with gout, including patients in our study, present with significant comorbidities such as diabetes, renal impairment, CVD and gastrointestinal disease [27]. Each of these comorbid conditions is associated with elevated levels of inflammatory cytokines [11] which in turn can affect both appetite and cause malabsorption syndromes know as a malnutrition-inflammation complex. This complex in previous studies has been reported in the past to effect lipid levels with the exception of HDL-C. This may help explain the findings in our study suggesting the comorbid conditions may have a stronger effect than hyperuricemia regarding HDL-C.

The novel findings emerging from this study should be considered within the context of some methodological limitations. Primarily, it was cross-sectional making causal inferences implausible. Secondly, although adherence to allopurinol was not tracked in this study, uric acid levels in some patients may not have decreased as expected due to lack of compliance with their allopurinol prescription. Among chronic diseases, gout has among the lowest rate of patient adherence to therapy [28]. Furthermore, hyperuricemia is associated with a variety of medical conditions, which could explain the varying outcomes associating uric acid and lipid levels [4, 16, 21, 25, 29]. Though allopurinol is used to control levels of uric acid in gout patients, a secondary benefit could be another means of controlling elevated levels of cholesterol which is a significant comorbid condition in gout patients.
